# A tragedy of the (academic) commons: interpreting the replication crisis in psychology as a social dilemma for early-career researchers

**DOI:** 10.3389/fpsyg.2015.01152

**Published:** 2015-08-06

**Authors:** Jim A. C. Everett, Brian D. Earp

**Affiliations:** ^1^Department of Experimental Psychology, University of OxfordOxford, UK; ^2^Department of Philosophy, Oxford Uehiro Centre for Practical Ethics, University of OxfordOxford, UK

**Keywords:** reproducibility of results, social dilemmas, methodology, best practices, psychological science

## Précis

Several proposals for addressing the “replication crisis” in social psychology have been advanced in the recent literature. In this paper, we argue that the “crisis” be interpreted as a disciplinary social dilemma, with the problem facing early-career researchers being especially acute. To resolve this collective action problem, we offer a structural solution: as a condition of receiving their Ph.D. from any accredited institution, graduate students in psychology should be required to conduct, write up, and submit for publication a high-quality replication attempt of at least one key finding from the literature, focusing on the area of their doctoral research. We consider strengths, weaknesses, and implementation challenges associated with this proposal, and call on our colleagues to offer critical response.

## The replication crisis as a social dilemma

Social dilemmas—situations in which collective interests are at odds with private interests—are an enduring feature of the modern world (Hardin, 1968). Social dilemmas have two fundamental characteristics: first, that each individual receives a higher payoff for defecting from what is in the collective interest (e.g., using all of the available resources for one's own advantage) than for cooperating, regardless of what other individuals do; and second, that all individuals are better off if they all cooperate than if they all defect (Dawes, 1980). A number of high-profile contemporary issues can be seen as social dilemmas, including the global energy crisis, preservation of the rainforests, and climate change. But social dilemmas can play out on a smaller scale as well, for example within an academic discipline. In our own discipline of social psychology, for instance, the ongoing “replication crisis”—see Earp and Trafimow (2015) for an overview and analysis—could be seen as stemming in part from just such a dilemma: that is, a conflict between self- and collective-interest.

To wit: while it is in everyone's interest that high-quality, direct replications of key studies in the field are conducted (so that one might know what degree of confidence to place in previous findings from the literature), it is not typically in any particular researcher's interest to spend her time conducting such replications. This is for a number of reasons: (1) such replications may be time-consuming; (2) they are likely to take energy and resources directly away from other projects that reflect one's own original thinking; (3) they are generally harder to publish; (4) even if they are published, they are likely to be seen as “bricklaying” exercises, rather than as major contributions to the field; (5) they (accordingly) bring less recognition and reward, including grant money, to their authors—and so on (see Earp and Trafimow, 2015, for further discussion).

In this brief commentary we reflect on our position as early-career researchers in the field of psychology facing a disciplinary social dilemma: How can we, and others in a similar position, do our part to conduct—and attempt to publish (see Earp et al., 2014)—high-quality replications of others' work, while simultaneously moving forward in our own careers by publishing novel and important findings?

Our focus on early-career researchers is deliberate. Compared with more established researchers—i.e., those with tenured positions at a university or similar—the problem we are describing is especially acute. Consider the case of an ambitious young scientist, who understands that she must “publish or perish.” Since replication studies are harder to publish in the first place, and are considered less prestigious if and when they do come out, she might reason that she does not have much of a choice here: as a function of time invested, every direct replication that she conducts (and attempts to publish) will reduce her chances, on balance, of achieving meaningful career security, compared against the other researchers on her “rung of the ladder” who decline to conduct such replications, and focus instead on their own novel experiments.

The stakes of this dilemma must not be understated. For an early-career researcher, failure to publish enough of the “right kinds” of studies (i.e., novel, “ground-breaking” work), in the “right” stretch of time (i.e., not too long after receiving one's Ph.D.), may result in failure to find work as a scientist. Thus, it is not just a matter of doing a little bit better for oneself, at the communal cost of contributing—by omission—to the crisis of replication; but at least potentially a matter of choosing between working as a scientist (at all), and being forced to give up and pursue a different career. How, then, might the replication crisis be addressed in a way that does not put early-career researchers in such an untenable position?

## The need for structural solutions

In their landmark paper, Messick and Brewer (1983) identify two types of solutions to social dilemmas: structural solutions and motivational solutions. Structural solutions are those that come about through coordinated and organized group action, and often involve regulatory agencies or socially approved coercion to constrain individual motivation in the collective interest. In contrast, motivational solutions rely on individual preferences and social motives, seeking to maximize those factors that influence individuals to act for the collective good. To help resolve this tragedy of the scientific commons, both structural and motivational solutions should be considered.

Motivational solutions are clearly important in principle, and they may work in practice for individual researchers. However, we believe that psychologists—both new and established—generally conduct their research because they have a genuine interest in advancing the science. In other words, we would be surprised to find out that (especially young) behavioral scientists just aren't *interested* in conducting high-quality replication studies, insofar as they understand that these are important for the scientific integrity of their field. Instead, it is much more likely that they would be hindered by a recognition that their doing so could put them at a distinct, even career-ending disadvantage compared to their peers who spend more time on original research. For this reason, structural approaches seem more likely to be successful in the long run as a way of resolving this crisis of replication.

A number of proposals for structural solutions have been offered. These include establishing specific forums in which to publish replications (Koole and Lakens, 2012), endorsing publication standards that require internal replications by the same lab that performed the original study (Roediger, 2012), and greater openness about data and methods (Wicherts et al., 2012). As Frank and Saxe (2012) note, however, most of these suggestions “are—at their core—requests for busy scientists to do something that is both less exciting and less rewarding than conducting new research” (p. 600).

In light of this observation, Frank and Saxe offer a suggestion of their own. What they argue is that “students in laboratory classes should replicate recent findings as part of their training in experimental methods” (p. 600). They go on share a personal experience: “in [our] own courses, [we] have found that replicating cutting-edge results is exciting and fun; it gives students the opportunity to make real scientific contributions (provided supervision is appropriate); and it provides object lessons about the scientific process, the importance of reporting standards, and the value of openness” (ibid).

We believe that Frank and Saxe (2012) are onto something important. But we have a number of concerns. First, they seem to be talking about undergraduate students, whose ability to conduct high-quality replications may be limited, at least with respect to certain kinds of studies. Second, the work of these undergraduates would have to be finished, most likely, during the window of a single term, which creates further limitations to this approach. And third, Frank and Saxe seem to present “teaching replication” as an option, which may simply re-introduce the problem of a social dilemma: those professors who opt-out of teaching replications would have more time for other projects.

## A new proposal

Considering both the strengths and potential weaknesses of Frank and Saxe's proposal, then, we asked ourselves: What about shifting the focus to graduate school? Imagine the following scenario: in order to receive a Ph.D. in psychology from any accredited institution in the United States (and perhaps in other nations as well), it is a requirement that one will have (1) conducted a high-quality “direct” replication of a major finding in their area (i.e., the area upon which their original doctoral research will be based); (2) written up the replication attempt to professional standards, no matter which way the data come out, and (3) made a good-faith effort to publish the paper in one of a growing number of high-quality online journals (such as *PLoS ONE*) that publish reports of well-conducted, valid experiments regardless of their novelty or their perceived “importance.”

This would address several potential problems associated with Franke and Saxe's (2012) suggestion, if it were to work as a structural solution to the replication crisis in psychology. First, Ph.D. students would be more likely, compared to undergraduates, to have the requisite skills (under the supervision of their advisors) to conduct truly high-quality replications. Second, they would not have to “cram in” a replication attempt in a single term, but could take as long as was necessary to do a good job, given the much higher stakes that would be involved (i.e., they could not be awarded a Ph.D. without meeting the above three criteria). And third, if it were a requirement of every accredited Ph.D. program, then no single graduate student could be at a disadvantage for conducting replications compared against her peers in other programs.

One implication of this approach is that it would remove the untenable burden from early-career researchers (post Ph.D.) to spend time on replication studies during a critical period of their career development, in a context in which, in any event, such a burden could not be uniformly enforced. Moreover, since all such researchers would already have contributed something meaningful toward the resolution of the replication crisis, they could feel free to spend their precious research hours on whatever projects they deemed to be most advantageous either personally or professionally. Finally, a new wave of Ph.D. students comes through each year: if all were required to conduct at least one replication study before graduating (according to the criteria specified above), this would represent a major, and constantly renewable, source of high-quality replications, focused on the most important and/or contentious studies at the cutting edge of psychological science.

## Implementation

How could such a requirement be established? While numerous practical difficulties would have to be addressed, at least some of the following steps seem clear: (1) the idea would have to be spread through the appropriate channels, and considered seriously by influential researchers; (2) it would have to gain widespread acceptance as a promising policy change, worth putting into practice, and (3) it would have to be raised in a decision-making context at the right level of authority such that it could be adopted and/or imposed simultaneously upon all accredited psychology programs nationally or internationally. How this would occur is by no means straightforward. But before any of the above steps could be taken, the basic soundness of the idea would have to have been established—which means subjecting it to critical scrutiny. Accordingly, we present it here in a public forum, and invite discussion and constructive feedback from our colleagues.

In the meantime, for those who do see some merit in our proposal (i.e., department chairs, members of faculty steering committees, Ph.D. supervisors, and other interested parties) it could perhaps be instituted on a voluntary basis, in a more piecemeal fashion. This would not, of course, fully address the collective action problem—at least not all-at-once—since students in replication-requiring programs would presumably have less time, all else being equal, for original research compared to their peers in other programs without the requirement. But it could nevertheless start a wave of momentum, by which the policy was adopted “from the bottom up” until it eventually reached a critical mass. This would be especially likely, it seems to us, if the early adopters were programs with already-outstanding reputations for research excellence and scientific rigor in training Ph.D.s, so that other programs would be encouraged to follow suit. Moreover, the graduates of these prestigious programs would have a number of compensatory advantages—due to the strong reputation of their affiliation during training—that could offset some of the loss in “original” research time due to the extra replication requirement. Although an imperfect solution, this would help address some of the potential bootstrapping issues associated with the persistence of the underlying social dilemma during the period of policy transition.

## Conclusion

It has been argued that science is a game involving “rules (not cheating), individual players (researchers), competing teams (paradigms), arbiters (reviewers and editors), and the winning of points (publications) and trophies (professorships, grants, and awards)” (Bakker et al., 2012, p. 1; see also Mahoney, 1976). We argue that for psychology to recover from the current crisis and emerge as an exemplar of behavioral science, it is not enough to merely change the motivations of the individual players and hope that they stick to the rules. Rather, for the game of science, the rules must be made clear—and enforced on a structural level.

Young researchers, with the very real pressure to publish original findings, and to secure research grants in order to sustain a living, pay a much higher cost by performing replications than the comparable cost paid by a tenured professor. At any given stage, the decision to expend one's time and energy on a replication study vs. a novel study is a zero-sum game: in choosing to do a replication, one forfeits that time for working on something else. And this commodity of time becomes more valuable as the stakes get higher: with a greater need to produce novel, high impact publications, the costs of time spent performing replications also increase. Therefore, we have argued that the burden should be removed from early-career researchers, and shifted onto graduate students as a condition of obtaining their Ph.D. Because this could in principle be uniformly enforced (i.e., through major accreditation bodies, or by mutual agreement among program leaders, working “from the bottom up”), it has the potential to eliminate any personal disadvantages that might accrue to individual students. It would represent, therefore, in our estimation, a powerful step forward in resolving the “replication crisis” in our field.

### Conflict of interest statement

The authors declare that the research was conducted in the absence of any commercial or financial relationships that could be construed as a potential conflict of interest.
